# The association between digitalization and mental health: The mediating role of wellbeing at work

**DOI:** 10.3389/fpsyt.2022.934357

**Published:** 2022-08-04

**Authors:** Jianmin Sun, Hongzhou Shen, Syed Ibn-ul-Hassan, Amir Riaz, Aura Emanuela Domil

**Affiliations:** ^1^School of Management, Nanjing University of Posts and Telecommunications, Nanjing, Jiangsu, China; ^2^Department of Commerce and Business, Government College University Faisalabad, Layyah Campus, Layyah, Pakistan; ^3^Department of Management Sciences, COMSATS University Islamabad, Lahore Campus, Lahore, Pakistan; ^4^Faculty of Economics and Business Administration, West University of Timisoara, Timisoara, Romania

**Keywords:** mental health, digital health, digitalization, job performance, IT infrastructure, well-being, IT proactive stance

## Abstract

The study aims to measure the mediating relationship of wellbeing at work between digitalization (IT infrastructure, IT business spanning, and IT proactive stance) and their effect on mental health. The study uses primary data collection techniques to gather data and used purposive sampling to analyze the data. The sample size of the study is 349 respondents. The research uses Smart PLS software to measure the relationship through bootstrapping and algorithms. The study finds a significant positive mediating role of wellbeing between digitalization (IT infrastructure, IT business spanning, and IT proactive stance) and their effect on mental health. The study outcomes are helpful for managers and policymakers.

## Introduction

In the past decades, technological advancement has profoundly altered global work practices with innovative developments. Significantly, the disruptive digital landscape has become the impetus for novel digitization, substantially overriding traditional digital tools with modern interventions. In recent years, the twenty-first-century modern technological revolution has developed promising digital capabilities to cover humans' personal and professional lives, potentially bringing positive health outcomes (1).

Digitalization is a novel phenomenon that has elevated the use of information technology across various domains. There has been strong demand for its application in the healthcare sector. However, in recent years, the mental health of frontline workers has not received adequate attention despite healthcare being a highly competitive industry in which employees are exposed to significant psychological challenges (2). As such, this literature states that as healthcare psychological problems are accelerating, it has become essential to focus on their impairing impact on employees' overall mental health (3, 4).

Mental health in relation to technology has become a prime interest of current researchers, with some researchers signaling the potential for the use of technology to considerably improve employees' mental health. While in recent years, technology adoption has been shown to bring promising health outcomes in many fields, unfortunately, the value of digital capabilities has not been realized in the healthcare industry (5). One study states that the emerging role of digitalization in the healthcare sector is vital, thus highlighting the need to realize and understand the association between IT capabilities and employees' psychological wellness (6).

The technological solutions implemented across medical domains (e.g., medicine and psychology) are of increasing interest to researchers in terms of novel innovations. Digitalization, a technology-driven notion, has diffused its characteristics into the origin of the healthcare sector. Novel IT capabilities have allowed healthcare organizations to adapt to changing working conditions, thus supporting employees' mental health (7). The real-time access afforded by IT has assisted the hospital industry by optimizing workflow. In fact, in healthcare, the accelerating technological advancement has garnered a tremendous boost to employees' wellbeing. Engaging and involving frontline technical staff in the design and rolling out of new IT infrastructure (ITI) has allowed organizations to understand the value of digital tools in improving employees' mental health.

The ITI alludes to organizations' digital assets such as tools, software, hardware, and applications (8). Significantly, ITI is recognized as a vital tool for fostering employees' mental health (9, 10). Technology is deeply rooted in the health economy, with IT business spanning (ITB) facilities impacting employees' mental health. As such, ITB refers to an organization's ability to adopt novel digital tools, therefore improving firms' operations (11). This novel business capability (i.e., ITB) helps organizations boost employees' mindfulness and productivity (12–14).

In particular, at present, an overwhelming number of technologies are available, meaning organizations need to understand the role of digital innovations in influencing employees' mental health. In this regard, the literature review provides evidence that to ensure the psychological needs of the frontline workers, health institutions should focus on developing technological capabilities for combating the progressing psychological vulnerabilities (15). Therefore, the IT proactive stance (ITP) has become prominent, whereby being forward-thinking about IT is used to achieve the business goal (16). Significantly, to achieve such goals, an organization must ensure its employees' healthy mindset. As a result, the ITP has emerged as a popular tool for ensuring employees' mindfulness.

Significantly, in recent years, occupational digital mental health has played a profound role in eradicating the potential barriers to achieving workplace wellness (17). Numerous factors contribute to elevating workplace problems. However, these increasing psychological vulnerabilities encourage medical institutions to mitigate health issues, thereby ensuring workplace wellbeing (15, 18). A healthy technological environment influences individuals' psychological health and, ultimately, workplace wellbeing and behavior. As a result of IT's increasing significance, organizations are adopting novel IT capabilities for ensuring wellness at work (19).

Undoubtedly, technological abilities have the potential as a global solution to growing psychological vulnerabilities. However, the healthcare sector demands that firms implement digitalization to gain greater attention in the coming decades (20). Technological health interventions provide numerous opportunities to combat the growing health crises. They maximize frontline technical support to establish a healthy environment. However, besides the influential role of technology, the literature shows that current employees are reluctant to adopt digitalization tools (21). In particular, one study states that this fear leads to health organizations lacking technological implementation, adversely influencing employees' psychological wellbeing (22).

However, against this drawback, this study demonstrates a novel conceptual model, presenting a systematic literature review on employees' mental health and workplace wellness. This study consolidates dominant factors that boost employees' mental and workplace wellbeing. Then, to reach a possible conclusion, the study highlights the role of digitalization (e.g., ITI, ITB, and ITP) in influencing employees' mental health. Moreover, it also sheds light on the effect of IT capabilities on workplace wellbeing. In the same vein, the study investigates the mediating role of the wellbeing at work nexus on digitalization and employees' mental health.

Significantly, this study promotes employees' mental health and wellbeing regarding digitalization. In particular, to the best of our knowledge, this study is pioneering in illustrating the role of IT capabilities (e.g., ITI, ITB, and ITP) in influencing employees' mental health. It explains a new concept that highlights the mediating role of wellbeing at work in this context. Therefore, on the scale of digitalization, this study presents valuable knowledge on employees' mental health and wellbeing. The study's findings are targeted toward researchers, policymakers, healthcare institutions, and the medical administration to suggest ways to improve employees' mental wellbeing.

This study comprises six different sections. The next section (i.e., “Literature review”) presents a conceptual model highlighting the study background. The “Methodology” section prescribes the methodological tools and techniques needed for study analysis. The “Results” section explains the analysis results, while the “Discussion” section discusses the significant study outcomes. Finally, the “Conclusion” section concludes the study by suggesting the research findings and implications.

## Literature review

### IT infrastructure and mental health

In recent years, the rapid advancement in digital technologies has altered the nature of work, thereby requiring employees to radically respond to the technological change. Among these developments, the ITI has emerged as an inevitable tool in assisting employees' workplace activities. The ITI has empowered workers to perform to their potential, thus bringing positive healthcare outcomes. In particular, this mental health innovation (e.g., ITI) is a convenient way of overcoming health crises in the workplace setting (17, 23). In recent years, technology has significantly evolved, bringing numerous opportunities for frontline workers. In explaining this notion, the literature states that, in healthcare, the high potential of digital technology fosters employees' psychological wellbeing and performance (10, 24).

In particular, workplace mental health is structured around modern digital developments. Novel IT innovations reduce the growing health ramifications, thereby engendering positive health outcomes (e.g., psychological wellbeing) (25). In fact, technology's rapid transformation of the world has worked as a catalyst, resolving problems across the healthcare ecosystem. As such, prior research states that digital tools have inevitably made healthcare organizations embrace novel technologies, thus promoting employers' positive psychological wellbeing (26). Overall, with the increasing significance of technology in healthcare, medical institutions should ensure proper utilization of technology to foster employees' mental health. Therefore, in light of the previous literature, the current study suggests the following hypothesis:

***H1****: IT infrastructure has a positive and significant impact on mental health*.

### IT business spanning and mental health

Mental health is a significant part of a person's wellbeing. With the growing number of individuals experiencing mental health crises, understanding the impact of technological change has become vital for ensuring individuals' psychological health. Technology integration helps organizations enhance employees' mental health. In the present digital era, IT-enabled advancements (i.e., ITB) are deeply rooted in firms' structures (27). Therefore, in ensuring positive mental health, ITB integrates preventive health technologies into the firm's structure (28, 29).

In particular, at present, the high pace of disruption (e.g., ITB) has updated and transformed firms' activities, shifting researchers' focus to mental wellbeing. The literature suggests that virtual IT platforms have made employees assess their psychological needs, thereby ensuring a higher degree of mindfulness (13, 30, 31). Those working in the hospital industry face high-level psychological issues (e.g., stress, anxiety, and depression). However, the rapid digital developments have profoundly altered the nature of the work, thereby combating the growing health vulnerabilities. As such, the ITB advances employees' mental health by mitigating the psychological risks associated with the workplace (32). Therefore, rather than just implementing technology, firms should build a clear understanding of its use for promoting employees' mindfulness. Consequently, based on the literature, this study proposes the following hypothesis:

***H2:*** *IT business spanning has a positive and significant impact on mental health*.

### IT proactive stance and mental health

In the digitization world, IT capabilities have brought numerous opportunities that support a workplace's mental wellbeing environment. Therefore, the workplace changes derived from the IT tools have encouraged the employees to learn novel tools for minimizing the effect of growing psychological vulnerabilities. As such, the literature states that owing to the effectiveness of the ITP, organizations should quickly respond to the changing workplace needs, optimally predicting the new opportunities (33, 34). In this regard, the ITP helps detect the employees' psychological needs, thereby illuminating the signs of health crises. The ITP provides opportunities to present organizations with critical information regarding their employees' health and wellbeing. Prior research shows that, at present, mental health technology enables organizations to respond to growing health challenges (35). Due to the increasing ITP role, the literature suggests making a high investment in disruptive technologies, thus ensuring positive health outcomes (i.e., psychological wellness) (36). Therefore, in light of the literature review, this study suggests the following hypothesis:

***H3:*** *IT proactive stance has a positive and significant impact on mental health*.

### IT infrastructure, IT business spanning, IT proactive, and wellbeing at work

#### IT infrastructure and wellbeing at work

In recent years, digitization and the prioritization of ITI have gained firms' attention, thereby nurturing employees' workplace wellbeing. In particular, current focus on employees' wellbeing has profoundly extended beyond just focusing on building a healthy workplace environment. ITI is crucial for maintaining optimal wellbeing (37). It increases employees' happiness and workplace wellbeing (38). Numerous tools have been found to support workplace wellbeing, with studies demonstrating that ITI works as an effective means to ensure employees' workplace wellness (39). Undoubtedly, predicting employees' workplace wellbeing has become the top priority of current firms. Due to information communication technologies (ICTs) growing relevance, the intensified role of ICT improves the employees' wellbeing at work. IT acceleration plays a critical role in managing workplace health issues. As such, the research shows that digital transformation focuses strongly on improving workplace wellbeing (40). In fact, ITI is critical to developing workplace wellbeing. Hence, given the literature review, this study proposed the following hypothesis:

***H4:*** *IT infrastructure has a positive and significant impact on wellbeing at work*.

#### IT business spanning and wellbeing at work

Undoubtedly, advancing globalization has allowed IT developments to open new avenues for improving workplace wellbeing. In the context of digital innovation supporting workplace wellness, one study showed that modern development had fostered a change in the nature of work, thus leading to the health interventions (e.g., ITB) to become the prime determinants of workplace wellbeing (41). The digitalization capability (i.e., ITB) supports the technology used in firms' practices. ITB is a disruptive technological model that has surprised researchers with its transformational aspects (for example, AI-based digital assistants) (42). The ITB capability nurtures the workplace environment by minimizing the workload. In recent decades, intense workloads have caused employees to face severe health repercussions, thus decreasing their workplace effectiveness. In explaining this notion, the study states that the ITB embedded in the firm's processes influences the employees' health, thereby shaping the workplace structure and work demand (43). In particular, technological advancement profoundly alters employees' workplace activities. The ITB elevating the technological change increases individual support for wellness. In this regard, the literature suggests that this novel innovation encourages management to realize the use of digitization to achieve workplace improvements (44). Hence, based on the prior literature, this study proposes the following hypothesis:

***H5:*** *IT business spanning has a positive and significant impact on wellbeing at work*.

#### IT proactive stance and wellbeing at work

Modern inventions have gradually become popular in ensuring workplace success. In recent years, digital-enabled wellbeing measures have been popularized as an integral initiative for raising awareness regarding wellbeing at work (45). Workplace wellness is a critical development that demands digital tools to improve employees' wellbeing. This technological capability provides solutions to the organization, thus encouraging a healthy workplace (46). The ITP renders tech support to the employees, potentially ensuring workplace wellness. The ITP supports effective IT programs for identifying the opportunities for achieving workplace growth. Additionally, it enhances the organization's internal environment by causing organizations to invest in workplace wellbeing. Prior research states that this health-increasing technology considerably satisfies organizations' needs for workplace wellness (47). These digital technologies minimize the negative impact of the growing workplace problems (48). Since technology has been applied to firms' structures in a widespread manner, it has become important for the organization to effectively utilize IT capabilities to combat workplace challenges, thus ensuring workplace wellbeing (49). Therefore, in light of the past studies, the following hypothesis is proposed:

***H6:*** *IT proactive stance has a positive and significant impact on wellbeing at work*.

## Mediating role of wellbeing at work

In the healthcare sector, workplace wellness supports employees' psychological health. Employees with good mental health assist organizations to cope with increasing workplace stressors, thus, in turn, ensuring the employees' wellbeing. As such, the research states that workplace wellbeing predicts employees' mental health (50). Mental health is a product of workplace wellbeing. However, understanding this phenomenon involves recognizing the worth of maintaining wellbeing at work. Healthy workplace activities manifest in workers' mindfulness. In this regard, research suggests that organizations tailor their workplace practices to engage individuals in wellness activities, thus facilitating employees' mental health (51). Therefore, at present, creating and building a healthier organizational environment is critical to achieving wellbeing. In explaining this notion, the research states that employees from different domains have recorded workplace wellness as a significant predictor of their mental health (52). Accordingly, this study proposes the following hypothesis:

***H7:*** *Wellbeing at work has a positive and significant impact on mental health*.

Undoubtedly, the massive shift toward technology has fostered numerous opportunities for organizations. It has highlighted how the workplace well-influences employees' mental health. Furthermore, it has been observed that the implementation of novel technologies in the workplace creates a positive wellness culture, thus supporting employees' wellbeing. Employees' mental health highly depends on workplace wellness. Accordingly, the research states that digital capabilities have gained significant popularity by scaling up employees' health outcomes by improving workplace wellbeing (53). In particular, the ITI ensures employees' positive mental health and wellbeing at work. Therefore, ITI increases workplace happiness and wellbeing by minimizing the growing workplace stressors (54). In fact, ITI is a novel phenomenon influencing employee mindfulness and wellness at work (55).

The increasingly demanding nature of work in many industries has led to calls for technology adoption to improve employees' wellbeing. At present, technological changes have enhanced global working conditions, improving the workers' health quality. Digital innovation fundamentally changes the work landscape, thereby yielding the positive effects of IT on employees' wellbeing. Prior research explains that the ITB capability narrows down the pathology of distress by inserting technology into the business process (56). In fact, such digitalization measures significantly enhance the workplace environment by boosting employees' mindfulness. Based on this statement, the research shows that the ITB promotes wellness at work, thereby the ICT aspect can be used to ensure psychological gratification (57). Consequently, to address mental health problems, organizations should consider the prevalence of novel digital measures (e.g., ITB) in ways that can promote work-life balance and positive psychological outcomes (19).

**Figure 1:**
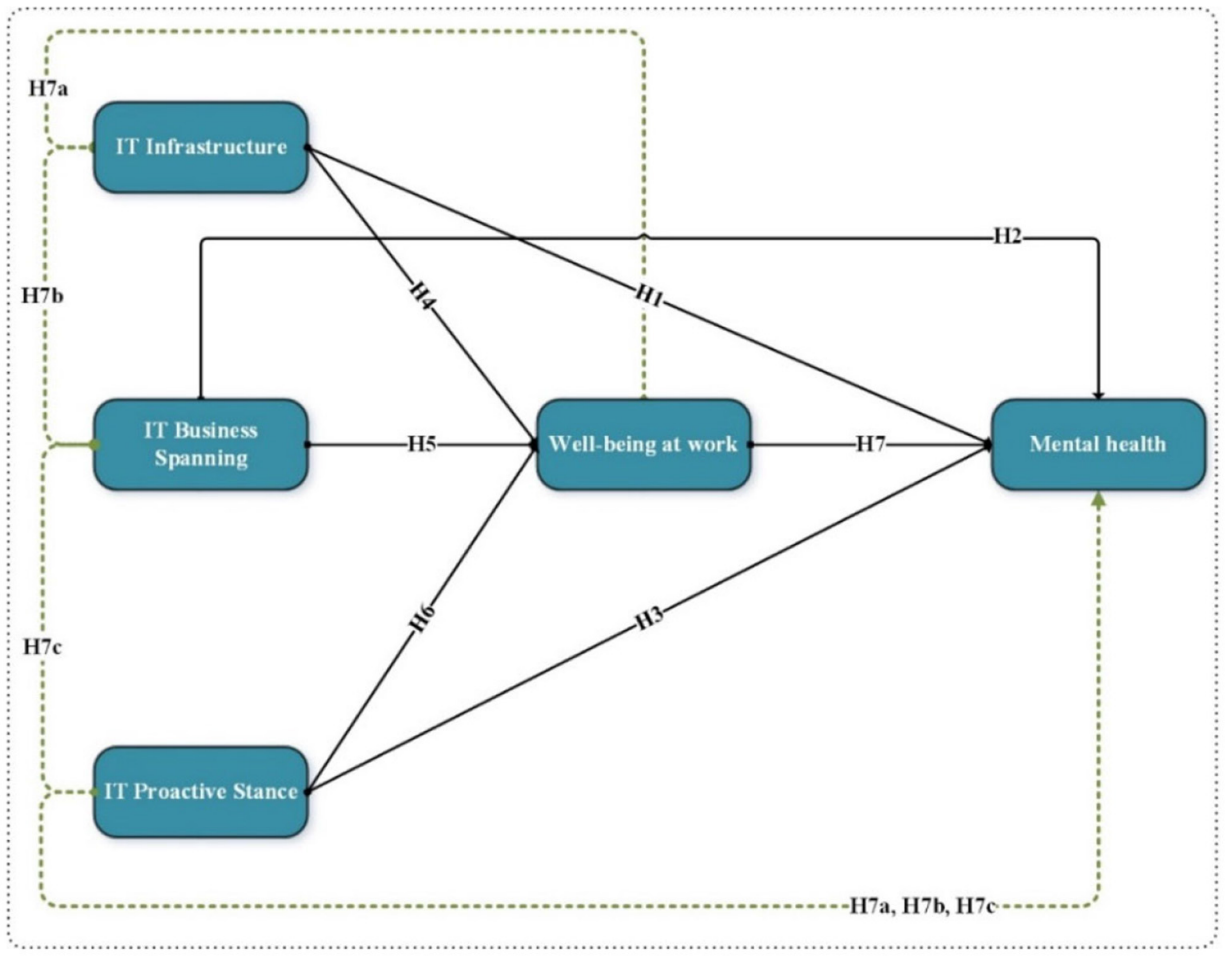
Conceptual framework.

In the present world, employees' wellbeing is a prominent focal point. In achieving this goal, IT capability has opened new avenues, with healthcare institutions being encouraged to embrace novel digital tools for promoting workplace wellness and influencing employees' mental wellbeing. At present, the technical intervention has assisted organizations to improve workplace wellness. In this regard, the ITP has become a dominant capability in fostering workplace wellness. Furthermore, building on this notion, the ITP innovations have helped companies to maintain a healthy working environment, thus boosting employees' mental health. In particular, this ICT measure has enabled organizations to establish a positive workplace atmosphere, thereby promoting employees' psychological wellbeing (47). Overall, digital health technology has contributed to enhancing employees' mental health and workplace wellbeing (19). Therefore, based on the data gathered, this study proposed the following hypotheses (refer to Figure 1):

***H7(a):*** *Wellbeing at work mediates the relationship between IT infrastructure and mental health*.
***H7(b):*** *Wellbeing at work mediates the relationship between IT business spanning and mental health*.
***H7(c):*** *Wellbeing at work mediates the relationship between IT proactive stance and mental health*.

## Methodology

The study's primary objective was to analyze the impact of digitalization on employees' mental health while considering the mediating role of wellbeing at work. The study has adopted a quantitative approach for the data collection. The data were collected from the employees working in the service sector of Pakistan. A convenience sampling technique was adopted, and data were collected through questionnaires. We have developed the questionnaire in dual languages (English and Urdu). Back-to-back translation for collecting data was used for “Urdu” language questionnaires as the data were collected from Pakistan, where the mother language is not English. We distributed 420 questionnaires among the employees from November 2021 to December 2021; 349 valid questionnaires were received from the participants. The Statistical Package for the Social Sciences (SPSS) and Partial Least Squares regression (PLS) were used for data analysis.

The measurement scale for the IT infrastructure, IT business spanning, and IT proactive stance consisted of three items and was adapted from the study of Nwankpa and Roumani (58). The sample items include “Data management services and architectures (databases, data warehousing, data availability, storage, accessibility, sharing, etc.),” “Developing a clear vision regarding how IT contributes to business value,” and “We are capable of and continue to experiment with new IT as necessary.” Wellbeing at work was measured on the nine-item scale adopted from the study of Demo and Paschoal (59). The sample items include “Over the past 6 months, my work made me feel proud” and “Over the past 6 months, my work made me feel active.” Employees' mental health was measured on the 5-item scale adapted from the study of Wu et al. (60). The sample item includes “I have everything to look forward to.”

This study applied the common method bias using Harman's single-factor approach. The variance extracted by one single factor is 10.435%, <50%, indicating no common method bias (61).

## Results

SmartPLS is used to analyze data in this study using the partial least square structural equation modeling (PLS-SEM) approach. This approach can examine complex models simultaneously, less restrictive to data assumptions. It can handle constructs with few measurement items compared to conventional approaches such as covariance-based SEM or multiple regression (62).

## Descriptive analysis

Descriptive analysis results relating to the study participants are reported in Table 1. These results show the frequencies and percentages of the study participants in terms of gender, age, highest qualification, and marital status.

**Table 1:** Descriptive statistics.

**Items**	**Frequency**	**(%)**
	**(*N =* 349)**	
**Gender**
Male	161	46.1
Female	188	53.9
**Age**
19–30	44	12.6
31–40	95	27.2
41–50	84	24.1
51–60	76	21.8
>60	50	14.3
**Education**
Intermediate	67	19.2
Bachelor	113	32.4
Master	124	35.5
MPhil/Others	45	12.9
**Marital Status**
Single	59	16.9
Married	290	83.1

## Measurement model

Reliability and validity of the measurements were first ensured through measurement model testing before testing the study's hypotheses. These results are reported in Tables 2, 3. The scores of factor loadings exceeded 0.65, and the average variance extracted (AVE) values were also above 0.50, as reported in Table 2. These factors' loading and AVE scores warranted the convergent validity of the study measures (63). Moreover, as reported in Table 2, the Cronbach's alpha (α) reliability score and composite reliability (CR) of all the variables well exceeded the cutoff value of 0.70 (64), establishing the reliability of the scales.

**Table 2:** Reliability and validity results.

**Construct**	**Items**	**Loading**	**α**	**CR**	**AVE**
IT infrastructure	ITI_1	0.737	0.847	0.848	0.583
	ITI_2	0.703			
	ITI_3	0.779			
	ITI_4	0.830			
IT business spanning	ITB_1	0.676	0.843	0.842	0.572
	ITB_2	0.779			
	ITB_3	0.733			
	ITB_4	0.830			
IT proactive stance	ITP_1	0.704	0.836	0.836	0.560
	ITP_2	0.787			
	ITP_3	0.781			
	ITP_4	0.719			
Well-being at work	WBW_1	0.784	0.929	0.929	0.592
	WBW_2	0.836			
	WBW_3	0.710			
	WBW_4	0.702			
	WBW_5	0.837			
	WBW_6	0.783			
	WBW_7	0.735			
	WBW_8	0.800			
	WBW_9	0.724			
Mental health	MH_1	0.758	0.877	0.877	0.588
	MH_2	0.728			
	MH_3	0.731			
	MH_4	0.825			
	MH_5	0.787			

**Table 3:** Discriminant validity results.

**Constructs**	**1**	**2**	**3**	**4**	**5**
1. IT business spanning	0.757	0.505	0.565	0.569	0.564
2. IT infrastructure	0.507	0.763	0.568	0.547	0.547
3. IT proactive stance	0.562	0.565	0.749	0.556	0.555
4. Mental health	0.570	0.548	0.559	0.767	0.563
5. Well-being at work	0.568	0.548	0.555	0.566	0.770

**Figure 2:**
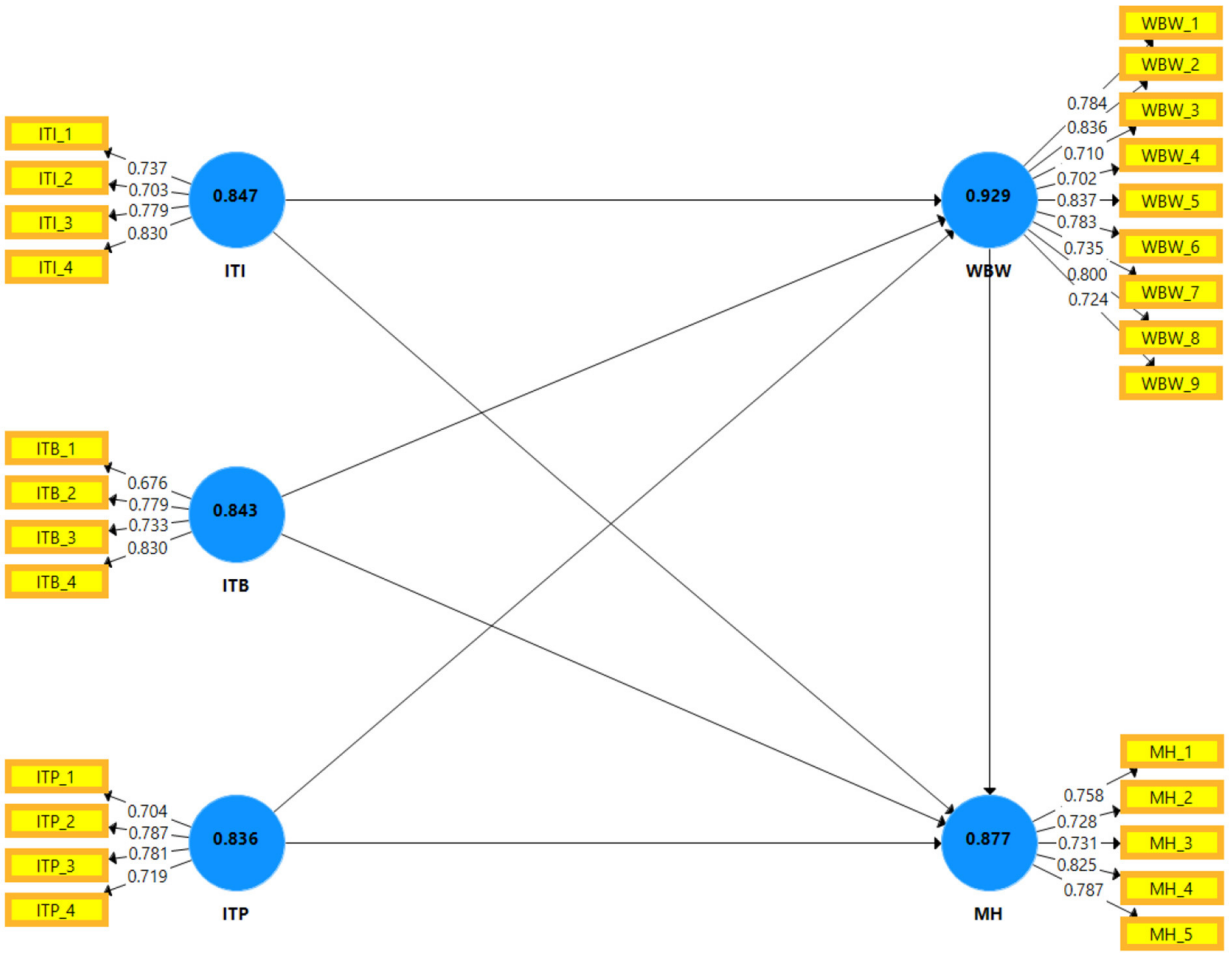
Measurement model PLS-SEM diagram.

In addition, Table 3 revealed the discriminant validity scores of the study variables using the Fornell and Larcker (65) criterion and a more robust and advanced approach named the heterotrait-monotrait (HTMT) ratio. According to Fornell and Larcker's (65) criterion, the square root scores of the AVE of each construct should be more than its correlation values, which was well achieved in the case of this study. Finally, the HTMT ratio score of the construct should be <0.85 to establish discriminant validity. Both these results ensured the discriminant validity of the study scales. Measurement model results are presented in Figure 2.

## Hypotheses testing

Table 4 presents the standardized path coefficient scores of all the hypotheses proposed in the direct relationships of the study (H1–H7). The relationship of IT infrastructure with mental health (β = 0.202^**^, *t* = 3.079), IT business spanning with mental health (β = 0.239^**^, *t* = 3.158), IT proactive stance with mental health (β = 0.192^*^, *t* = 2.642), IT infrastructure with wellbeing at work (β = 0.26^***^, *t* = 4.186), IT business spanning with wellbeing at work (β = 0.302^***^, *t* = 4.390), IT proactive stance with wellbeing at work (β = 0.239^***^, *t* = 3.267), and wellbeing at work with mental health (β = 0.213^**^, *t* = 3.279) were found to be significant, statistically. Overall, the above results statistically supported this study's first seven hypotheses (H1–H7).

**Table 4:** Results of direct effects.

**Hypothesis**	**Direct relationships**	**Std. *Beta***	**Std. error**	***T*-values**	***P-*values**
H1	ITI → MH	0.202	0.066	3.079	^**^
H2	ITB → MH	0.239	0.076	3.158	^**^
H3	ITP → MH	0.192	0.073	2.642	^*^
H4	ITI → WBW	0.26	0.062	4.186	^***^
H5	ITB → WBW	0.302	0.069	4.390	^***^
H6	ITP → WBW	0.239	0.073	3.267	^***^
H7	WBW → MH	0.213	0.065	3.279	^**^

* *p < 0.05*,

** *p < 0.01*,

*** *p < 0.001*.

**Figure 3:**
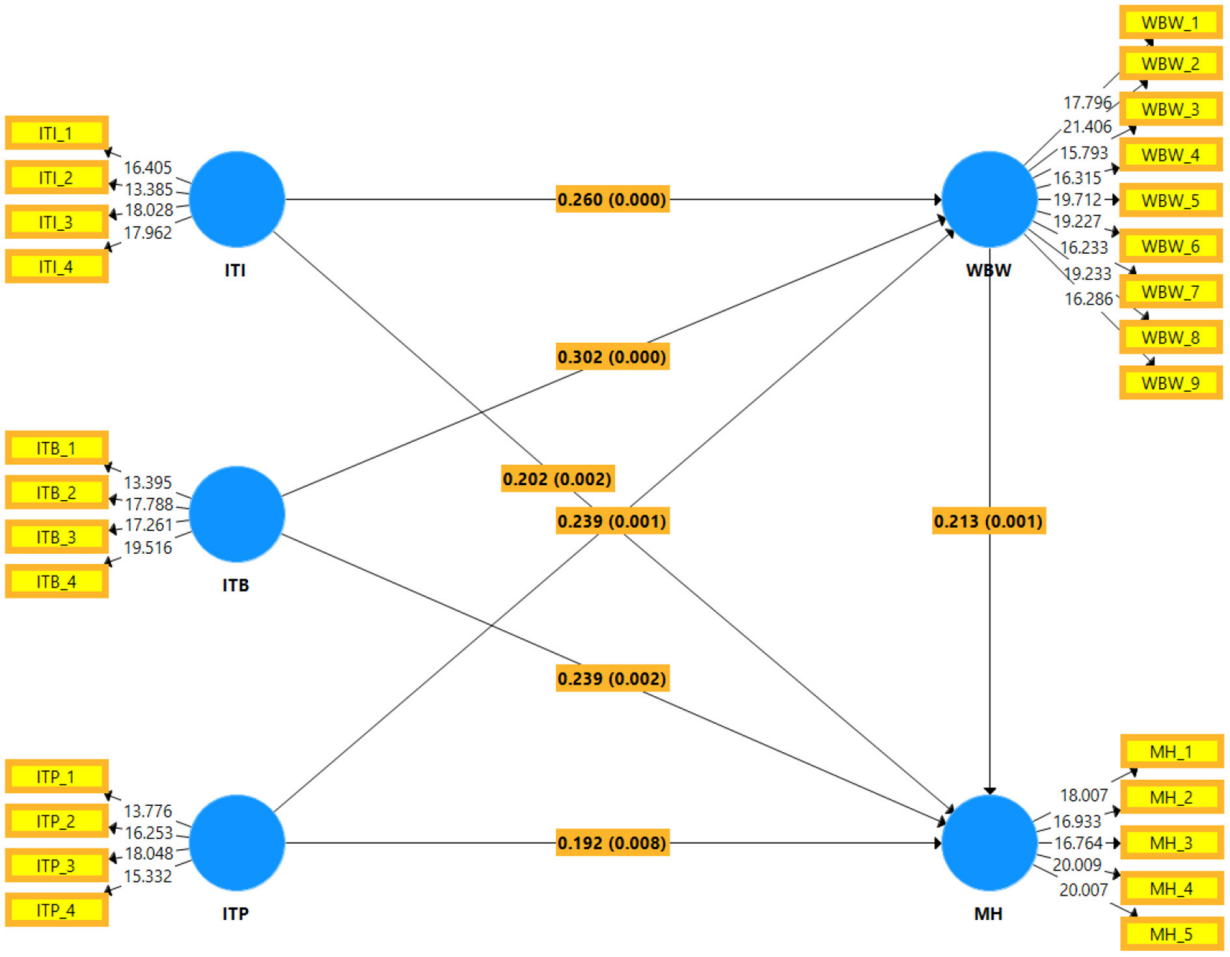
Structural model PLS-SEM diagram.

Furthermore, the last three hypotheses (H7a-c) claimed that wellbeing at work mediates the relationship of IT infrastructure, IT business spanning, and IT proactive stance, respectively, with mental health. First, as reported in Table 5, wellbeing at work mediated the relationship between IT infrastructure and mental health (β = 0.055^*^, *t* = 2.224), empirically supporting the H7(a) of the study. Next, as claimed in the H7(b) of the study, the mediating role of wellbeing at work for the relationship between IT business spanning and mental health (β = 0.064^*^, *t* = 2.481) was also empirically supported. Finally, wellbeing at work also mediated the relationship between IT proactive stance and mental health (β = 0.051^*^, *t* = 2.133) and got empirical support for the H7(c) of the study. Figure 3 shows the structural model analysis results.

**Table 5:** Results of mediation effects.

**Hypothesis**	**Indirect relationships**	**Std. *Beta***	**Std. error**	***T*-values**	***P-*values**
H7a	ITI → WBW → MH	0.055	0.025	2.224	^*^
H7b	ITB → WBW → MH	0.064	0.026	2.481	^*^
H7c	ITP → WBW → MH	0.051	0.024	2.133	^*^

* *p < 0.05*.

## Discussion

In recent years, novel technological advancement has significantly enhanced employees' mental health irrespective of the industry. IT plays an essential role in ensuring workplace wellness and employees' mental health. As such, many organizations have invested in digital solutions for expanding their services. Organizations are increasingly realizing the value of modern advancements. With the technical developments, healthcare employees have found support to offer best practice care, thereby empowering their mental health and wellbeing at work. In explaining this phenomenon, this section explores the effect of digitalization on employees' mental health and wellbeing at work, considering the previous literature findings.

Traditionally, digital innovations have mainly been the focus on researchers, but now organizations are placing more emphasis on technology as a fundamental part of their structures. The research suggests that technological infrastructure supports employees' mental health (66). ITB has emerged as a solution to resolving employees' mental issues, with one study stating that technology integration (i.e., ITB and ITP) has enabled organizations to modify business activities, thereby ensuring positive mental health (32). Moreover, the growth in ICT has led IT capabilities to emerge as novel tools for fulfilling employees' psychological demands. In fact, our study's findings agree with the previous literature, thereby accepting H1, H2, and H3.

Technology has often represented a disruptive yet positive force in its impact on workplace wellness. IT can be used to solve workplace problems by effectively allowing the institution's technical capability to enhance workplace wellbeing (67). The use of health technology infrastructure has increased in recent years. Prior research shows that good ITI decreases psychological vulnerabilities, ultimately facilitating employees' workplace wellbeing (68). As the global health crisis increases, ensuring workplace wellbeing has become necessary for organizations in this sector. In this regard, a growing body of research states that digital health inventions (i.e., ITB and ITP) bolster employees' workplace wellness (69). In explaining this notion, the existing literature states that to gain long-term benefits in the form of employees' mental health, organizations should create a healthy working environment, thus supporting workplace wellness (70). In fact, our research findings also revealed the same results, thus verifying the research assumptions made in H4, H5, and H6.

Digital technological innovation has brought numerous benefits to healthcare organizations, by facilitating workplace wellbeing and employees' mental health. As such, technology adoption in the healthcare sector reflects the notion that modern tools influence workplace wellbeing (71). In particular, digitalization (e.g., ITI, ITB, and ITP) plays a significant role in enabling positive outcomes in this sector. The findings reveal that digital health capabilities have become increasingly popular as a means to provide employees with a good quality of living (i.e., mental health), thereby supporting their wellbeing at work (68). Hence, our study also supports the prior literature, substantially accepting H7 (a, b, and c). To sum up, our research findings support the previous literature, reiterating the view that technology adoption in the healthcare sector has a positive outcome in terms of employees' mental health.

## Conclusion

Undoubtedly, over the years, an overwhelming number of digital innovations has led employees to integrate the digital tools that create a healthy psychological work environment. Studies show that a poor workplace environment leaves a heavy toll on employees' mental health. Accordingly, to understand the effect of digitization on mental health in the workplace, this study presented a systematic review of how digitalization capabilities in healthcare influence employees' psychological health. The study explored the relationship between IT capabilities and employees' mental health. It drew a link between digitization approaches and employees' mental health concerning the mediating effect of wellbeing at work.

Good IT practices are essential for fostering employees' wellbeing. Our research findings indicate that IT capabilities (e.g., ITI, ITB, and ITP) positively influence employees' mental health and workplace wellbeing. A review of the literature revealed that the technology deployed in the healthcare sector has improved employees' mental health and wellbeing. Altogether, our results are positive, thus supporting the previous studies. In fact, the findings of this study open pathways for future researchers, policymakers, and healthcare institutions, by directing their focus on studying technology and mental health toward the healthcare sector. This study holds valuable knowledge for healthcare organizations regarding the need for a focus on employees' mental health. In fact, the findings of the study are significant in terms of identifying digitization as supporting employee psychological wellness. It recommends that policymakers in healthcare should consider how technology can improve employees' workplace wellbeing and mental health to gain positive health outcomes.

## Author contributions

All authors listed have made a substantial, direct, and intellectual contribution to the work and approved it for publication.

## Funding

The authors acknowledge financial support from the National Natural Science Foundation of China (Grant No: 71974102) and the Philosophy and Social Science Fund of Tianjin City, China (Grant No: TJYJ20-012).

## Conflict of interest

The authors declare that the research was conducted in the absence of any commercial or financial relationships that could be construed as a potential conflict of interest.

## Publisher's note

All claims expressed in this article are solely those of the authors and do not necessarily represent those of their affiliated organizations, or those of the publisher, the editors and the reviewers. Any product that may be evaluated in this article, or claim that may be made by its manufacturer, is not guaranteed or endorsed by the publisher.
